# Teenage Pregnancy: Impact of the Integral Attention Given to the Pregnant Teenager and Adolescent Mother as a Protective Factor for Repeat Pregnancy

**DOI:** 10.1100/tsw.2007.12

**Published:** 2007-02-09

**Authors:** Maria José Carvalho Sant'Anna, Kepler Alencar Mendes Carvalho, Amanda Melhado, Verônica Coates, Hatim A. Omar

**Affiliations:** ^1^ Adolescent Clinical Unit, Department of Pediatrics, Faculty of Medical Sciences Santa Casa, São Paulo, Brazil; ^2^ Department of Pediatrics, University of Kentucky, Lexington, USA

**Keywords:** pregnant adolescents, repeat pregnancy in adolescents, protection factor, integral support for the pregnant adolescents, Brazil

## Abstract

The purpose of this study was to evaluate the impact of the integral attention to the health of pregnant adolescents and adolescent mothers, having follow-up from the Integral Support Program for the Pregnant Teen (ISPPT), with the intention to determine quality of life and prevent repeat pregnancy. A prospective study comprised 85 adolescents attended by the ISPPT between January 2002 and June 2006 who participated in meetings during pregnancy with a multidisciplinary team that provided orientation concerning family planning, self-esteem, pregnancy prevention, motivation to continue education and/or work, and evaluate the postpartum mother-child relationship. The following were analyzed: education level, marital status, contraceptive use, thoughts and attempts at abortion, repeat pregnancy. This study was approved by the Human Research Ethics Committee. The Epi-Info v6.0b software was used for data and result evaluation using the means and the chi-squared test. The mean age of the adolescents was 15.7 years, 3.52% had repeat pregnancy within a mean follow-up of 23 months after childbirth, the mean education level was 8.1 years, 30.5% dropped out of school, with 79.4% occurring before pregnancy, 64.6% used no contraceptives, 68.3% were single, and 81.3% had a positive role model. One year after birth, 67.5% studied, 50% worked, 55.1% lived with the partner, 77% correctly used contraceptives, every child lived with their mothers and their vaccinations were up to date. The results demonstrate that the global attention given to the health of adolescent mothers and pregnant adolescents is a protective factor for pregnancy relapse and quality of life.

## INTRODUCTION

Even though generalizations concerning the sexual behavior and fecundity of teenagers/adolescents cannot be made, the factors associated with pregnancy during this developmental period have been discussed throughout the world. Among the biological factors, the ever more premature onset of puberty and menarche has predisposed to early sexual behavior. Emotional blocks can occur during this period and the most important are the magical thoughts of "this will never happen to me", the confirmation of their fertility, aggression from the parents, and the guilt or hope and the desire to become a mother. These aspects in association with low self-esteem, family relationship problems, emotional distress, lack of knowledge about contraceptive measures and their limited use, as well as sociocultural changes and the accelerated urbanization that took place in the last 4 decades, have led to elevated pregnancy rates in adolescent girls.

Regarding the beginning of one's sexual life, several Latin American studies have verified that women with little education begin their sexual life earlier than those with more education. Adolescents that have little or no emotional support, whether from the presence of conflict in the family or from absent parents, in association with the significant lack of access to knowledge concerning sexuality and reproductive health from the family, school, and health services, present a greater tendency to have less plans and expectations in relation to their education and careers, and are more vulnerable to the risk factors associated teen pregnancy[1].

Despite the fact that teenage pregnancy is frequently related to socioeconomic disadvantages, we need to consider that it occurs within the context of lack of opportunity and limited life options, and is marked by interruptions in the individual's formal education[2]. Frequently, pregnancy is wished for, but not planned. For these girls, despite its early occurrence, pregnancy is desired and may be the only chance for a change in their status in society; it is possible they believe that it is the only option available for them to become a part of a social group. One study reported[3] that the percentage of pregnant teenagers that actually married their children's fathers was lower than that of adult women and usually ended up in divorce[4].

The difference concerning the number of pregnant teens among developed countries is mainly determined from sex education and the effective use of contraceptive measures, rather than sexual behavior[5]. Despite the 30% decrease in teen pregnancy rates between 1991 and 2003 in the U.S.[6], it still has the highest incidence among the industrialized countries, having 820,000 unwanted/undesired pregnancies per year[7,8]. In Brazil, the National Research on Demographics and Health of 1996 revealed that there was a 30% reduction in fecundity in all age groups, with the exception of the adolescent age group[9]. The number of births in the adolescent population corresponds to roughly 10% of all of births worldwide per year; in Brazil, the number of newborns from adolescent mothers corresponds to 26.75% of births, having regional variations, with the highest rates occurring in the north and northeast[10], excluding the intentional abortions, which occur illegally and whose rates are unknown. In the survey conducted by the IBGE in 2000, it was demonstrated that the rate of fecundity between the ages of 10 and 14 years increased 108% in the last 10 years[10].

In the U.S., the prevalence of a repeat pregnancy in the year subsequent to giving birth was estimated at 20%[11]. According to the data provided by the SEADE[12], in 2002, the state of São Paulo had a 22.9% rate of repeat pregnancy during adolescence. Rigsby et al.[13] found rates ranging between 30 and 50% in the absence of puerperal orientation. From the national literature, Takiuti[14] observed a rate of 30.5% in São Paulo within a 2-year follow-up period; Melhado et al.[15] also found high rates. Teenage women are at higher risk for repeat pregnancy when they are under 16 years of age and their sex partner is over the age of 20 years, when they do not study or have an educational setback, and who do not have a stable relationship with their child's father[16].

## ISPPT — Integral Support Program for the Pregnant Teenager

The presumption that pregnancy is a disaster during adolescence brings about the fantasy that it will always determine serious risks to the mother and child. It has been clearly proven that adolescents who receive support from a multiprofessional team during pregnancy and after their child's birth have better organic and psychosocial results during and after pregnancy, and enhance her identification as a woman.

Based on these data, the ISSPT was created in the Clinic for Adolescents of the Pediatrics Department of the Santa Casa de São Paulo in 1985, with the goal of providing integral and global support to aid these young women in dealing with the biopsychosocial risk factors involved in this population. This program provides open-group discussions on a bimonthly basis with a team composed of pediatricians, social workers, psychologists, physical therapists, the pregnant teenagers, their partners, as well as adolescent mothers that participated during their pregnancy along with their children. These reunions, which last approximately 2 h, represent a great opportunity to aid the teen during her pregnancy, childbirth, and maternity. The group discusses the organic and psychosocial factors involved, to give the young couple and their family a foundation on which to cope with the situation. Certain factors are prioritized, such as health, the development of maternity and paternity, stimulation of the individual identity of the young couple, issues concerning family life, valorizing self-esteem, motivation to return or continue their formal education or careers in order to provide the tools necessary to avoid staying in poverty, child care issues, breastfeeding, and prevention of a repeat pregnancy during adolescence. The adolescents who participate show great interest in the program, and demonstrate interest and confidence concerning their future.

Considering all of these factors, pregnancy during adolescence has become an important issue for the specialists involved in the area, and several studies and programs have been and are being conducted with the aim of reducing its incidence and recurrence. This is a problem that society, health professionals, and political figures must face head on. The importance of the present study is to provide the instruments for the qualification of the ISPPT as a protective factor for repeat pregnancy during adolescence, to improve quality of life and health of the mother and the child.

### Purpose

This study aims to evaluate the impact of the integral attention given to pregnant teens and adolescent mothers having follow-up with the ISPPT in the Clinic for Adolescents of the Pediatrics Department in a teaching hospital. In addition, we hope to characterize the situations that make the teenager more vulnerable to an early pregnancy and repeat pregnancies, and to establish strategies that will help to improve the program as a whole.

## METHODS

This is a prospective study comprised of 85 pregnant teenagers under the age of 18 years that participated in the ISPPT in the Clinic for Adolescents of the Pediatrics Department of the Santa Casa de São Paulo, Brazil, between January 2002 and June 2006, with the aim of promoting good self-esteem, orientation about reproductive health, and information about pediatric follow-up of their child.

Open-group meetings were held on a bimonthly basis with a team that included pediatricians, social workers, psychologists, physical therapists, pregnant teenagers, their partners, and teen mothers that participated during their pregnancy along with their child. These group meetings had the goal of preparing these young women for pregnancy and childbirth, improving family planning, encouraging self-esteem, and motivating them to return or continue education and careers, breastfeeding, childcare, and prevention of repeat pregnancies while still teenagers. After delivery, the mother and child have a monthly follow-up in the pediatric walk-in clinic during the first year, every 4 months during second year, and twice a year during the third year. They receive integral attention regarding health, self-esteem, sex education, and pediatric care.

The following variables were analyzed: age during first pregnancy, interval between first and second pregnancy, prenatal care, postpartum contraceptive orientation, age during the first sexual relationship, contraceptive method used, educational level, school attendance, marital status, relationship with the child's father, desire and planning for pregnancy, thoughts and attempts at suicide. This research was approved by the Ethics Committee on Human Research of the institution.

The Epi-Info v6.0b software was used for statistical analysis using the obtained means and the chi-squared test was applied to determine the difference between ratios.

## RESULTS

The patients were between the ages of 11 and 18 years (incomplete until conception), with a mean of 15.7 years, a mean of 14.5 years during their first sexual relation, and a median of 6 months between the beginning of their sex life and pregnancy. The mean duration of formal education was 8.1 years, 69.5% attended school, and, among those that abandoned school, 79.4% had done so before their pregnancy.

The majority of the teenagers (68.3%) that attended school were single, 19.8% lived with their parents, 48.2% with their partner, 54.9% came from families without a paternal figure, and 9.8% did not have contact with the child's fathers. A positive role model was found in 81.3% of the women who participated and it was either the mother or sister in 47.7% of the cases.

The partner of these women had been their first in 74.4% of the cases, and they had a mean age of 20 years, 86.4% worked, the majority (71.65%) did not attend school, even though they did present a good level of education (8.1 years). In 90.1% of the cases, it was their first pregnancy and roughly one-third (35.8%) of the women wanted the pregnancy, and almost all of the women registered their child (90.1%) (Table 1).

**TABLE 1**
**Relationship Between Contraceptive Methods Used by Adolescents Before and After ISPPT**

| Variables | Before (%) | After (%) | *p* Value |
|---|---|---|---|
| Use of contraceptive methods | | | |
| Yes | 35.4 | 77 | <0.05* |
| No | 64.6 | 23 | |
| Correct use of the contraceptive method | | | |
| Yes | 27.6 | 98.7 | <0.05** |
| No | 72.4 | 1.3 | |

* Chi-squared test ($X^2$); ** Fisher's exact test.

Despite the fact that the pregnancies were not planned in 93.9% of the cases, 35.4% used a contraceptive measure and among those that reported such use, only 27.6% did so in a correct manner. One year after giving birth, 77% used a contraceptive method, 98.7% correctly, presenting statistical significance in relation to the use prior to attending the ISPPT. We found that only 7.4% reported constant condom use, 32% reported never having used condoms, 60.5% used condoms on a nonregular basis, 20.7% of the women considered abortion, and 5% actually attempted abortion.

In evaluating the women 1 year after childbirth, 67.5% studied, 55.1% lived with their partner, all of the women lived with their child, and all had their children's vaccinations up to date. The mean duration of breastfeeding was of 5.25 months, with a median of 6 months.

The mean follow-up of the mothers was of 23.4 months and, in this period, only 3.52% of the women had repeat pregnancy during adolescence, with a mean time between the two deliveries of 22 months. The women that presented with repeat pregnancy were younger (mean of 14.8 years) in comparison to the other women, had the same partner, all lived with their mothers, they never met their father, and had not planned the second pregnancy. Of these women, 66.6% remained in school, they did not work, and they lived with their partners.

## DISCUSSION

This study used a sample population in which generalizations concerning the analyzed variables cannot be made. However, our results may be helpful for our understanding of the questions concerning sexuality during adolescence, with greater emphasis on pregnancy prevention and its early reoccurrence.

Among the risk factors associated with teen pregnancy and repeat pregnancy during adolescence are early sexual behavior, low level of education, dropping out of school, malstructured family, and lack of knowledge and use of contraceptives. Coard et al.[17] demonstrated that young women were beginning their sexual life ever earlier, which was cited as a risk factor for pregnancy and its recurrence during adolescence. In the present study, the mean age of women during their first pregnancy was 15.7 years, reinforcing that impact of early sexual activity demonstrated by Sant'Anna et al.[18]. Among the adolescents that had formal education, the mean age during the first sexual relation was 14.6 years, the mean time elapsed between the first sexual relation and pregnancy was 7.9 months, with a median of 6 months. Karofsky et al.[19] found a strong relationship between early sexual activity, unstructured families, and the lack of dialogue between the teenager and the parents. The influence of the family in the sexual behavior of young women has been analyzed under several aspects, with the familial context having a direct relationship with the initiation of sexual activities[17]. A positive role model was found in 81.3% of the cases, demonstrating the importance of a familial reproductive history. Ellis et al.[20] demonstrated that single-parent families, usually constituted by the mother, are associated with early sexual behavior and pregnancy during adolescence.

The mean time of education was 8.1 years at the time of pregnancy, which is high when compared to the national Brazilian mean, which is 5.4 years for the same age[10]. This result could be explained by the fact that the women involved in the study lived in a dense metropolitan area and also looked for assistance, similar to women with greater education and with greater motivation concerning their health. The lower number of children is directly associated to the mother's level of education, therefore, this factor determines greater reasoning and thinking about reproduction. Data from the Census of 2002[10] show that young women who reside in more developed urban areas have greater access to school. The majority of the women (69.5%) were in school when they first got pregnant and among those that had dropped out of school, 79.4% had done so before becoming pregnant, as has been found in other studies conducted in our service[18]. In general, it is considered that the women who abandon school have less chances of getting jobs. One year after childbirth, nearly two-thirds remained in school and one-half worked, which could be explained by the assistance from the ISPPT, which emphasizes self-esteem and interest in the future. Not returning to school 6 months after childbirth is considered a risk factor for repeat pregnancy during adolescence by the American Academy of Pediatrics[4].

We found that 93.9% had not planned pregnancy, however, 47.6% wanted to become pregnant, which reveals the lack of family planning that is common in this age range. However, it is a mistake to assume that every pregnancy during adolescence in unwanted since many of the women look to pregnancy as away to change their lifestyle, without, however, having awareness to the future consequences of a premature pregnancy; only 6.1% of the women planned their pregnancy in our study.

Despite pregnancy having not been planned in the majority of the cases, an elevated rate of unprotected sex was observed; only 35.4% of the women used contraceptive methods before pregnancy and of those that did, only 27.6% used it correctly, rates similar to those found by Sant'Anna et al.[18]. In evaluating the women 1 year after childbirth, 77% used contraceptive measures, 98.7% correctly, differences that are statistically significant. Studies have shown that the majority of young mothers use contraceptive methods, usually hormonal, in the 6 months following childbirth when compared to women who are not pregnant or have not given birth[21], a rate that declines with passing of time, with much lower values when evaluated 12 months after birth[22]. Despite this decline, adolescent mothers still present higher rates of hormonal contraceptive use than adolescents who have not given birth[22]. In an extensive review of the literature, Meade and Ickovics[23] found oral contraceptive discontinuation rates varying between 48–66% in 6 months and 68–73% in 12 months. With the ISPPT, we observed a high maintenance rate of hormonal contraceptives after 12 months since childbirth, reinforcing the importance of the program as a protective factor against repeat teenage pregnancy.

When specifically questioned as to the use of condoms, almost one-third had never used the method, highlighting the fact that condom use varies according to the significance of the affective sexual relation, thus making this theme of prevention only associated with access to information. In evaluating condom use, 7.4% reported their use, 32% never used them, and 60.5% used them sometimes. Kershaw et al.[22] did not find significant differences in relation to condom use before and after pregnancy, or among adolescent women who never became pregnant. Meade and Ickovics[23] observed that the majority of teens practiced unsafe sex before, during, and after pregnancy. Over half of the teenagers between 15 and 19 years of age are sexually active, yet only 75% use contraception during their first sexual relation and less than 30% regularly use contraceptive methods[24]. In Brazil, according to the PNDS, in 1996, only 14% between the ages of 15 and 19 years used some type of contraceptive[9].

The role of the teenager's partner should not be forgotten. Unfortunately, the father frequently does not admit responsibility for the pregnancy and usually leaves the pregnant teenager. Sant'Anna and Coates[25] found that 50% of the women did not have a relationship with the father when they first looked for prenatal assistance, with the support from the father being considered a strong protective factor during the pregnancy progression and childbirth. Among the adolescents in the present study, 55.1% of the mothers lived with the child's father and 26.3% did not have any contact with the father. Every mother lived with the child, who presented up-to-date vaccinations; the mean time of exclusive maternal breastfeeding was of 5.25 months, median of 6 months, reinforcing the capacity of these women to maintain their life goals, of being good mothers when receiving orientation about breastfeeding, proper child care, and the value of self-esteem.

Despite being illegal in Brazil, the option of abortion seems to be well disclosed among adolescents. We observed that 20.7% of the women considered abortion and 5% attempted abortion without success. According to Meyrick[26], research about sexuality in Latin America identifies pregnancy as the most-feared repercussion from sexual relations among teenagers, especially because of the financial consequences involved. Therefore a tendency exists for a higher propensity for abortion among adolescents than older women[26]. Such information corroborates the affirmation that adolescents constitute a risk group, considering that abortion is illegal in Brazil and is one of the main reasons for hospitalizations from infections, not to mention maternal death. Abortion represented 16% of all maternal deaths in women between the ages of 15 and 24years in the poorer regions of the country.

In this study, we discovered a 3.52% repeat pregnancy rate in adolescents having follow-up by the ISPPT, having a mean follow-up of 23 months, a value that is considerably low when compared to the rates from the state of São Paulo 22.9%[12]. Historically, repeat pregnancies during adolescence have been high, 22% of adolescents have a second child during the first year after postpartum and 25–50% during the second year[27]. Pfitzner et al.[16] found a recurrence of 10.6% within a 21-month follow-up period in a multidisciplinary program; in 1999, more than 20% of American adolescents presented with a second pregnancy within 2 years from the first[11], while Raneri and Wiemann[28] found 42.2% of repeat pregnancy within 24 months. When analyzing the small number of repeat pregnancies in our group, the women that did become pregnant again were younger, with a mean age of 14.8 years, and all maintained a stable relationship with the same partner, factors which were also found by Pfitzner et al.[16]. The adolescents that had repeat pregnancies all came from single-parent homes, all lived only with their mother, who also became pregnant during adolescence; 1 year after the birth of the first son, 66% were in school, none worked, or correctly used a contraceptive method. Among the protective factors for the second undesired pregnancy, the American Academy of Pediatrics[4] and Meade and Ickovics[23], in an ample review of the literature, considered that the return to school and postpartum contraceptive orientation were the most efficient in conjunction with use of condoms and hormonal contraceptives.

Unfortunately, our study has some limitations, such as a small sample size. We also found some problems in forming a control group due to ethical reasons, and the limited socioeconomic status of some of our patients, who frequently move and do not adhere to our service. Thus, we decided to compare our results with those present in the literature. The following factors also present some limitations: pregnancy rates are difficult to establish in Brazil due to the fact that abortion is illegal, thus it is done secretly. We believe that these limitations do not impact the main conclusion of our study, which is the protective effect of the ISPPT on repeat teen pregnancy, in their quality of life, in the abandonment of school, and in the preservation of a life project. We emphasize that pregnancy during adolescence, in addition to being a problem for the trajectory of a young woman's life, is also a social problem, taking into account the precarious conditions of our health services and from the probability that the pregnancy will give way to an abortion done in unsafe conditions. We would also like to emphasize the importance of the multiprofessional care for the global assistance to the pregnant adolescent; of the sexual orientation that may help young women to better get to know themselves and assume their own sexuality with appropriate decisions regarding contraceptive use, which reduces the risk factors.

## CONCLUSIONS

The results found suggest that the ISPPT serves as a protective factor against repeat pregnancy during adolescence, helps to improve self-care and quality of life of these teenagers, and in reduction of school drop out and maintenance of a life project. Adolescents who became pregnant for the second time were all younger than the other patients and none worked, and that the integral attention to the adolescent's health through a multidisciplinary team promotes improved self-esteem and responsible sex life.

## ACKNOWLEDGMENTS

The authors thank CNPq – Conselho Nacional de Desenvolvimento Científico e Tecnológico – "National Counsel of Technological and Scientific Development" for their generous financial support of this work. We are grateful to the Support Center for Scientific Publications of Santa Casa de São Paulo – Faculty of Medical Sciences for the editorial assistance.
